# Mr. Jackson, on the Cataract

**Published:** 1808-02

**Authors:** Thomas Jackson

**Affiliations:** Stonesby, Leicestershire


### *To the Editors of the Medical and Physical Journal.*

GENTLEMEN,

THE Cataract being a disease deeply affecting the comfort and happiness of mankind, has naturally attracted the attention of surgeons, from a very early period of time. And, certainly, when we compare the science and improvements of the present day, with the wild conjectures and coarse operations of our forefathers, we have ample reason to congratulate ourselves on our great increase of knowledge. But great as it comparatively is, there is too much cause to lament that we are not yet arrived at perfection; else why do we see some authors recommending the operation of extraction, and others of depression; some positively affirming that complete absorption of the lens will take place; others as positively affirming that it will not? May we not say, with a late celebrated author, "multum adhuc restat operis, multumque restabit; nec ulli nato post mille secula præcludetur occasio aliquid adhuc adjiciendi *."

As the operation of couching or depression is almost universally allowed to be attended with less risk, difficulty, and pain, than extraction, it is, without doubt, a matter of great importance to adopt it, provided it be found equally efficacious. No prudent man would hesitate to choose that operation, which would effect the intention with the greatest ease and safety, even though it might require more time for its completion. "Minima de malis," is an axiom not to be controverted. How far the needle may answer in membraneous cataract, farther experience must determine; but if we look at the many excellent cases of our countryman Mr. Hey, and also of Professor Scarpa, we shall see great reason to think, that it may be rendered pretty generally successful. With respect to its occasioning the absorption of the corpus chrystallinum, or lens (independently of every other consideration), the following case strikes me as being so pointed, that I think it my duty to communicate it. And although I am well aware that it is only one, of many cases of the kind, that have occurred, yet, as a relation of it may tend to corroborate the body of evidence in favour of the needle, and

* See Pott's Preface [illegible] [illegible]marks on Cataract.

thereby to diminish the pain necessarily attendant on the very nice and difficult operation of extraction, I trust I need no other apology for its insertion.

### Case.

Mrs. Elizabeth Gould, of Chadwell, aged 60, had the misfortune to have cataracts in both eyes: the cataract in the left eye had been formed two years; that in the right, three months. On inspecting the eyes, the pupils quickly dilated and contracted, according to the different degrees of light to which they were exposed. She was able to distinguish bright colours when presented to her, and had an imperfect glimpse of the outlines of large bodies that crossed her, which she described as shadows passing by her; but was incapacitated from following her accustomed occupations, or even going about the house, without feeling her way with her hands.

On the 12th of October, 1805, I couched her left eye, using Mr. Hey's needle; and had the satisfaction to find, as soon as the lens was displaced, and before the needle was withdrawn, that she saw my face. I then disengaged the needle from the lens, and passing its point through the pupil (that I might be quite sure that the fore and convex part of the capsule was fully and largely lacerated), withdrew it. For fear of irritation, the eye was closed immediately after the operation, and a proper bandage applied. Very little pain or inflammation followed the operation; but on examining the eye on the third day after, I was much chagrined to observe that the lens had risen into its natural situation. I had, however, the consolation to find my patient not altogether disappointed; as she perceived the light much more strongly than before the operation, and could also distinguish the outlines of objects in the room pretty well. Upon a more close examination of the eye, I perceived that the needle had pierced the lens in its centre; and had left a small perforation, through which she could see a little, when the pupil was somewhat dilated. This being the case, I concluded to wait some time, to see what absorption would effect; but had the mortification to find, that the degree of sight gained by the operation gradually decreased. And in about six weeks after the operation, she could see very little better than before it was performed. It appeared to me that the lens, by being divested of its capsule, and surrounded by the aqueous humour, had swollen; and that the enlargement of that body, body, had nearly closed the opening made into it by the needle. Thus situated (as the eye was free from inflammation), I determined on repeating the operation; and, on the 23d of November following, I again introduced the needle, but could not depress the lens, the needle passing through it in every direction; and it was evident to me, that the lens had acquired much softness from the first operation. I, therefore, endeavoured to assist the process of its absorption, by breaking down its texture; and dividing it into several pieces, withdrew the needle. This operation was also followed with little pain or inflammation; and, after a few days, I had the pleasure to find that she could distinguish objects tolerably correctly, when placed in certain directions. At this time, secondary or irritative inflammation * came on, notwithstanding which her sight rapidly improved; and, in the space of six weeks from this last operation, every particle of the lens was absorbed, her sight being sufficiently good for her to execute her domestic employments without glasses; and, with the assistance of a convex pair (at 2½ focus), she was able to thread and use her needle, and to read in a very small print.

My attention was now turned to the right eye. Reflecting on the circumstances that attended the recovery of the left, I determined on just introducing the needle, and simply to pass it through the lens, without endeavouring to depress it; leaving it to the chance of absorption. I was the more induced to follow this method, as I considered, that although it might require much time, it would be of no great consequence, as my patient could see well enough, with her recovered eye, to do any thing she found necessary.

On the 15th of March, 1806, I introduced the needle into the right eye; and passing it freely through the lens, withdrew it. I would here remark, that the lens appeared to me to be of a firmer consistence than that of the left eye. The eye was rendered rather turbid by the operation, but remained very easy until the sixth day, when very considerable inflammation came on, attended with much pain in the eye, and corresponding side of the nose; which continued during a month (notwithstanding my best endeavours to counteract it), and then disappeared, leaving

---

* Inflammation coming on at an uncertain period, long after that from the operation has ceased, and evidently occasioned by the lens itself.

the eye clear, and pretty free from pain; but no perceptible progress towards absorption had taken place.

The eye continued until the June following (three months) in this state, when she thought she could perceive more light; and on an attentive survey of the eye, I could distinguish a small crack or fissure in the body of the lens, where the needle had passed. From this time to the month of June 1807, a space of a year and a quarter from the operation, her sight kept gradually improving, and at that time was become pretty good, the lens continuing to waste, having the appearance of a rugged rock that was crumbling away, and seemed ready to be precipitated to the bottom of the eye. At this juncture secondary inflammation took place, and continued for about a month, when all of the diminished lens, that could be seen, was a very small fragment, laying at the bottom of the eye, behind the iris, and between it and the ciliary processes. This, however, was no impediment to vision. A very minute particle was discernible about a month ago, (a year and three quarters from the operation) but I understand is now hardly visible; and I am happy to add, that the sight of this eye is stronger and more perfect than the left, although it has been observed, that the degree of vision in that was quite sufficient for the common purposes of life.

Having stated the case, I shall conclude with a few practical remarks upon it, which appear to me not altogether unimportant.

First, I would observe, that with respect to the consistence of the cataracts, that although (as has been stated) they suffered the needle to pass through them, that neither of them were what have been termed soft. It is much to be doubted whether there are any cataracts so hard as entirely to resist the pressure of the needle, if not unskilfully managed, particularly when so fine a one as Mr. Hey's is used. That gentleman tells us, that in most of the operations he has performed, he has found the needle to pass through the lens in every direction; and I am ready to acknowledge, that (in the cases just related) both cataracts might possibly have been depressed in the first instance by a more experienced hand. This appears pretty evident from the length of time required for the absorption of the lenses, especially that of the right eye, which is not yet totally removed, although it is not to be doubted that it would have vanished long ere this, had a second operation been performed similar to that on the left eye.

Secondly, that the lens of the right eye appearing to be of of a firmer consistence than the left, seems to favour the opinion of Mr. Pott, viz. that it is probable that a diseased lens is never of so firm a consistence as one in a healthy state, and that consequently the more complete the disease, the nearer the lens will approximate to the soft state.

Thirdly, that the operation on the right eye being attended with more inflammation than the left, might be occasioned by its being performed in too early a stage of the disease, the operation itself being more simple than that on the left. There is no doubt, that (in a sound and perfect state of vision) a circulation is kept up between the corpus chrystallinum and the other humours of the eye; and probably as the disease advances, the vessels of circulation become impervious, by which means the circulating medium is cut off, the chrystalline itself becoming as an extraneous body; and, consequently, its dislodgement is likely to be followed with a less degree of inflammation.

Fourthly, that the sight of the right eye being more perfect and strong than the left, might arise from the more slow absorption and removal of the lens, by which means the loss of so important a body to the economy of the eye would be less missed, and vision ultimately be rendered more perfect; or it may be, that the operation being more simple than that of the left eye, that less organic injury took place, and consequently the structure of the eye would be left more perfect after the disappearance of the lens.

Fifthly, that in both eyes, after secondary inflammation came on, the business of absorption was greatly forwarded, and that probably secondary inflammation always takes place from the enlargement and consequent pressure of the lens, acting as an irritating cause to the eye. Farther, that the enlargement of the lens is the first step to its subsequent diminution, and final obliteration.

Sixthly and lastly, I would observe (as indeed is pretty evident from the case itself) that both eyes were recovered, not from depression, but absorption of the chrystallines, and that consequently, although in the operation of couching, the depression of the chrystalline is the primary object, yet if we should fail in this intention, that sooner or later (provided the capsule be properly and sufficiently lacerated) absorption will take place, and vision be restored, which it is humbly conceived this case will tend to illustrate. I am, &c.

THOMAS JACKSON.

